# Src Mediates Epigallocatechin-3-*O*-Gallate-Elicited Acid Sphingomyelinase Activation

**DOI:** 10.3390/molecules25225481

**Published:** 2020-11-23

**Authors:** Motofumi Kumazoe, Mai Kadomatsu, Jaehoon Bae, Yushi Otsuka, Yoshinori Fujimura, Hirofumi Tachibana

**Affiliations:** Division of Applied Biological Chemistry, Department of Bioscience and Biotechnology, Faculty of Agriculture, Kyushu University, Fukuoka 819-0395, Japan; obc2509jmk@yahoo.co.jp (M.K.); m2.erci.ai@gmail.com (M.K.); baejae8@yahoo.co.jp (J.B.); otsuka1014@gmail.com (Y.O.); fujimu@agr.kyushu-u.ac.jp (Y.F.)

**Keywords:** Src, EGCG, anticancer effect, FAK, sensing molecule, 67LR, cancer, cGMP, green tea

## Abstract

Epigallocatechin-3-*O*-gallate (EGCG) is one of the major bioactive compounds known to be present in green tea. We previously reported that EGCG shows selective toxicity through activation of the protein kinase B (Akt)/cyclic guanosine monophosphate (cGMP)/acid sphingomyelinase (ASM) axis via targeting its receptor 67-kDa laminin receptor (67LR), which is overexpressed in cancer. However, little is known about upstream mechanisms of EGCG-elicited ASM activation. In this study we show that the proto-oncogene tyrosine-protein kinase Src, also known as c-src, plays a crucial role in the anticancer effect of EGCG. We showed that EGCG elicits phosphorylation of Src at Tyr 416, a crucial phosphorylation site for its activity, and that the pharmacological inhibition of Src impedes the upstream events in EGCG-induced cell death signaling including upregulation of Akt activity, increase in cGMP levels, and activation of ASM. Moreover, focal adhesion kinase (FAK), which is involved in the phosphorylation of Src, is colocalized with 67LR. EGCG treatment enhanced interaction of FAK and 67LR. Consistent with these findings, pharmacological inhibition of FAK significantly neutralized EGCG-induced upregulation of Akt activity and activation of ASM. Taken together, FAK/Src play crucial roles in the upstream signaling of EGCG.

## 1. Introduction

Multiple myeloma is one of the hematologic cancers that develop in mature B cells [1]. Several prominent advances have been made in chemotherapy for multiple myeloma, and overall survival has been extended from 3 to 6 years in the past two decades [2]. However, multiple myeloma is still a difficult hematopoietic malignancy because of its chemoresistant character and because patients with multiple myeloma are typically older and adverse effects induced by anticancer agents pose difficulties in treatment.

Green tea is the extract of the leaves of *Camellia sinensis* and is one of the major beverages consumed globally. Several epidemiological studies indicate negative correlation between green tea consumption and the risk of cancer. For instance, Naganuma et al. demonstrated that the multivariate-adjusted hazard ratio of hematologic malignancies for individuals consuming 5 cups/day or more of green tea compared with those consuming less than 1 cup/day was 0.58 (95% confidence interval: 0.37, 0.89) based on a survey of 41,761 Japanese adults aged 40–79 years [3]. Consistent with these findings, Takada et al. showed that the values for hazard ratio for all hematologic cancers in people who drink green tea ≤2, 3–4, and ≥5 cups every day were 0.65 (0.42–1.00), 0.73 (0.47–1.13), and 0.63 (0.42–0.96), respectively compared with the people who never drink green tea [4].

Green tea intake has also showed beneficial effects in patients with oral cancer [5], colorectal adenoma [6], prostate cancer [7], and early chronic lymphocytic leukemia [8]. In the phase 2 clinical study on patients with chronic lymphocytic leukemia, Polyphenon E^TM^ containing 60% EGCG, the first botanical drug approved by the US Food and Drug Administration for the treatment of patients with external genital and perianal warts, showed potent anticancer effects in 29/42 patients with chronic lymphocytic leukemia without causing sever adverse effects [8].

Epigallocatechin-3-*O*-gallate (EGCG) is one of the major bioactive compounds in green tea and shows several anticancer properties [9,10,11] through interact with specific targets [12] and its pro-oxidative action [13]. We previously identified the 67-kDa laminin receptor (67LR) as the drug receptor of EGCG with Kd value of 40 nM [14]. Interestingly, 67LR the receptor of EGCG is highly overexpressed in several types of cancers including bile duct carcinoma [15], colorectal carcinoma [16], cervical preneoplastic lesions and neoplastic squamous cells [17], breast cancer [18], and melanoma [19] compared with their normal counterpart. Moreover, Shammas et al. showed that overexpression of 67LR in multiple myeloma cells plays a crucial role in selective toxicity of EGCG [20]. Consistent with their findings, we and another group showed overexpressed 67LR mediated selective toxicity in several types of hematopoietic cancer [21,22,23]. Moreover, we demonstrated that 67LR activated the protein kinase B (Akt)/endothelial nitric oxide synthase (eNOS)/cyclic guanosine monophosphate (cGMP) axis [24,25,26,27] as an early molecular event in EGCG-induced cell death. We also demonstrated that EGCG-elicited cGMP upregulation induced acid sphingomyelinase (ASM) activation through phospholipase C/protein kinase C delta systems [28]. However, little is known about the upstream mechanisms involved in EGCG-induced Akt activation.

In this study, we show that the proto-oncogene tyrosine-protein kinase Src also known as c-src an important kinase in cancer growth [29,30] plays a crucial role in upstream mechanisms involved in EGCG-induced Akt activation in multiple myeloma cells. Pharmacological inhibition of Src neutralized EGCG-induced Akt activation and cGMP upregulation. Consistent with these findings, EGCG induced the phosphorylation of Src at Tyr 416, a crucial phosphorylation site for its activity.

Moreover, focal adhesion kinase (FAK), which is involved in the phosphorylation of Src, colocalized with 67LR, and EGCG treatment enhanced the interaction of FAK and 67LR. These data indicated that FAK/Src might act as a part of the early mechanism after EGCG binding to 67LR.

## 2. Results

### 2.1. Pharmacological Inhibition of Src Attenuated Cell Death Inducing Effect of EGCG

**Figure 1:**
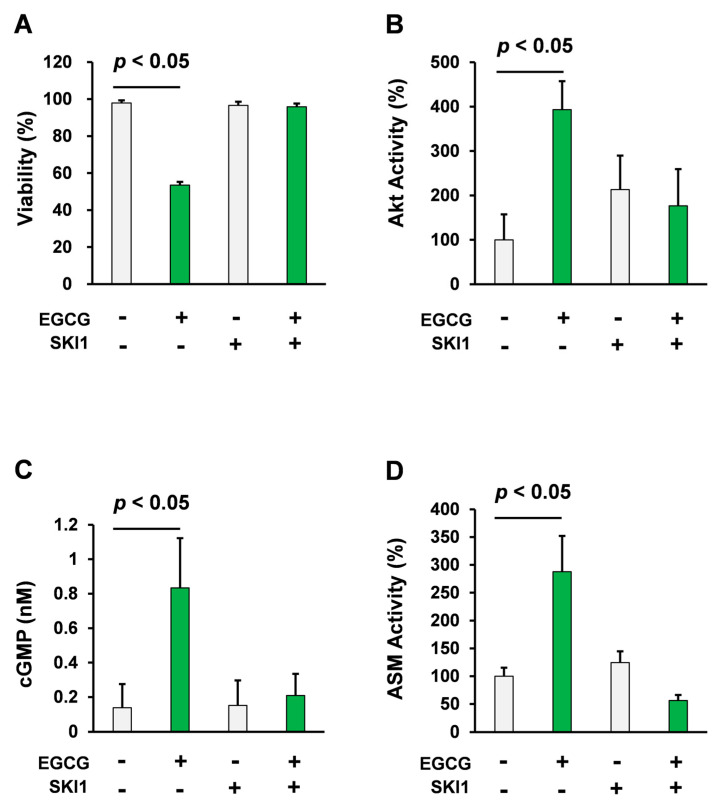
Pharmacological inhibition of Src attenuated the cell death inducing effect of EGCG. (**A**) U266 human multiple myeloma cells were treated with EGCG (10 μM) and SKI1 (2.5 μM, from 1 h before EGCG treatment) for 72 h. Cell viability was measured using the trypan blue method (*n* = 4). (**B**) U266 human multiple myeloma cells were treated with EGCG (10 μM) and SKI1 (2.5 μM; from 1 h before EGCG treatment) for 1 h. Akt activity was evaluated using the K-LISA kit (*n* = 4). (**C**) U266 human multiple myeloma cells were treated with EGCG (10 μM) and SKI1 (2.5 μM; from 1 h before EGCG treatment) for 3 h. cGMP levels were evaluated using competitive immunoassay (*n* = 4). (**D**) U266 human multiple myeloma cells were treated with EGCG (10 μM) and SKI1 (2.5 μM; from 1 h before EGCG treatment) for 3 h and ASM activity was evaluated (*n* = 3).

Consistent with previous reports [21,25], treatment with 10 μM EGCG decreased viability of U266 human multiple myelomacells. In contrast, pretreatment with 2.5 μM SKI1, a Src inhibitor, significantly attenuated EGCG-induced decrease in viability of U266 human multiple myeloma cells (*p* < 0.05, Student’s *t* test) as shown in Figure 1A.

We have previously reported that Akt activation is a crucial mechanism in the EGCG-induced cell death signaling pathway in multiple myeloma cells [25]. Moreover, compounds that enhance EGCG-mediated induction of Akt, enhance beneficial effects of EGCG [31,32]. Taken together, Akt might play a crucial role in the effect of EGCG. Consistent with our previous findings [25], EGCG treatment significantly upregulated the activity of Akt in U266 cells. In contrast, pretreatment with 2.5 μM SKI1 completely abolished the effect of EGCG on Akt activity (*p* < 0.05, Student’s *t* test) as shown in Figure 1B.

cGMP is an essential mediator of the beneficial effects of EGCG including its anticancer effect and anticancer stem cell effects [25,33,34,35]. Pharmacological inhibition of soluble guanylate cyclase diminished the effect of EGCG [25]. To assess the role of Src in the effect of EGCG on intercellular cGMP levels, U266 cells were pretreated with the Src inhibitor. The Src inhibitor significantly diminished the cGMP inducing effect of EGCG in this model (*p* < 0.05, Student’s *t* test, Figure 1C).

ASM is the downstream effector of EGCG-induced cell death in multiple myeloma cells [21,28]. Knockdown or pharmacological inhibition of ASM diminished the susceptibility of multiple myeloma cells to EGCG [21,28]. We also showed that cGMP induction is sufficient to induce activation of ASM [28]. Consistent with our previous findings, pharmacological inhibition of Src abolished ASM activation induced by EGCG (*p* < 0.05, Student’s *t* test, Figure 1D).

Taken together, Src plays a crucial role in EGCG-induced activation of the Akt/cGMP/ASM signaling pathway.

### 2.2. EGCG Induced Src Phosphorylation of Tyr 416 in Multiple Myeloma Cells

**Figure 2:**
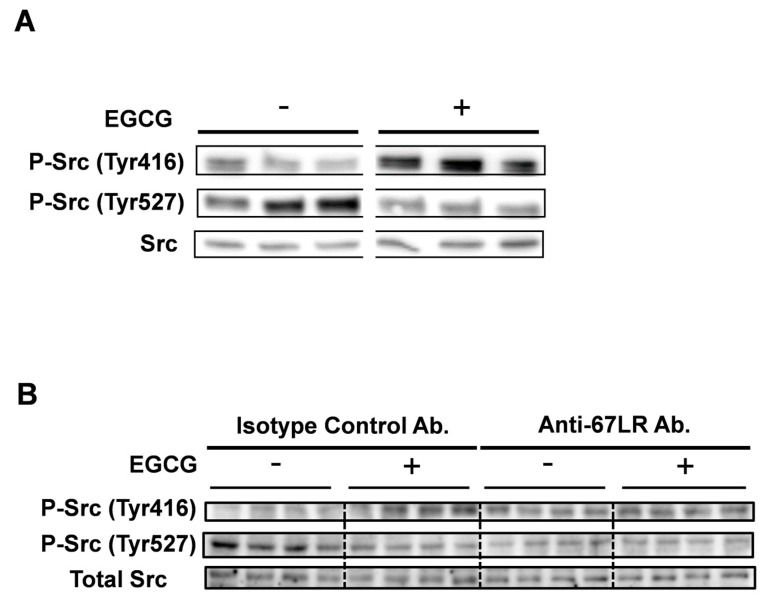
EGCG induced phosphorylation of Tyr 416 in multiple myeloma cells. (**A**) U266 human multiple myeloma cells were treated with EGCG (10 μM) for 30 min. Phosphorylation levels of Src were evaluated using Western blotting (*n* = 3). (**B**) U266 human multiple myeloma cells were pretreated with the anti-67LR antibody or with the isotype control antibody and treated with EGCG (10 μM) for 30 min. Src phosphorylation levels were evaluated using Western blotting (*n* = 4).

The activation of Src is regulated by phosphorylation; Tyr 416 is phosphorylated upon activation of Src [36]. Moreover, Fujii et al. reported that Src mutants with mutation of Tyr-416 to Phe showed lower promoter activation [37]. We hypothesized that EGCG treatment regulates Tyr 416, the crucial phosphorylation site of Src. In order to evaluate the effect of EGCG on this phosphorylation site, human multiple myeloma cell line U266 cells were treated with EGCG (30 min, 10 μM) and Tyr-416 phosphorylation levels were analyzed using Western blotting. EGCG treatment significantly increased phosphorylation of Tyr 416 the crucial site for Src activity (Figure 2A).

67LR is the receptor of EGCG and we previously reported that EGCG activated Akt through a 67LR-dependent mechanism [25]. In order to assess the role of 67LR in EGCG-induced phosphorylation of Src, U266 cells were treated with EGCG (10 μM) with or without of anti-67LR antibody for 1 h.

EGCG treatment increased phosphorylation levels of Src at Tyr 416 and that EGCG-induced phosphorylation was neutralized by pretreatment with anti-67LR antibody (Figure 2B). Taken together, EGCG elicited phosphorylation of Tyr 416 the crucial site for Src activity through 67LR dependent mechanisms.

### 2.3. Pharmacological Inhibition of FAK Attenuated the Akt/ASM Axis Elicited by EGCG

FAK is the kinase that is involved in integrin, the receptor of laminin dependent signaling [38]. As 67LR is a non-integrin laminin receptor and FAK is involved in Src activation [39], we hypothesized that FAK is part of the upstream mechanism of 67LR-dependent signaling.

**Figure 3:**
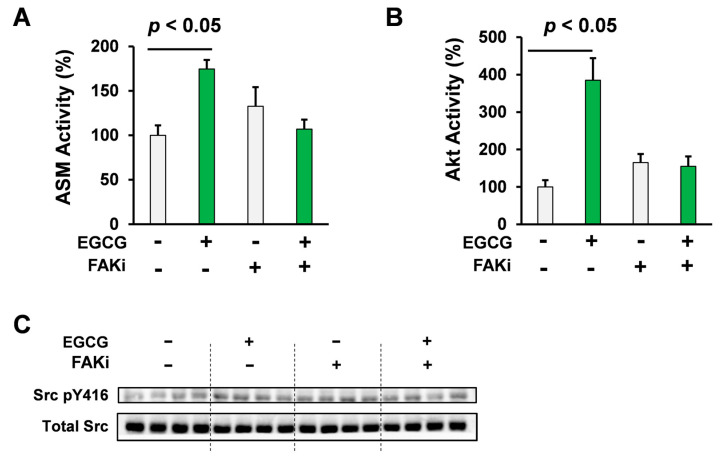
Pharmacological inhibition of FAK attenuated the Akt/ASM axis elicited by EGCG. (**A**) U266 human multiple myeloma cells were treated with EGCG (10 μM) and PF-573228 (1 μM; from 1 h before EGCG treatment) for 3 h (*n* = 3). (**B**) U266 human multiple myeloma cells were treated with EGCG (10 μM) and PF-573228 (1 μM; from 1 h before EGCG treatment). Akt activity was evaluated using the K-LISA kit (*n* = 3). (**C**) U266 human multiple myeloma cells were treated with EGCG (10 μM) and PF-573228 (1 μM; 1 h before EGCG treatment) for 30 min. Src phosphorylation levels were evaluated using western blotting (*n* = 4). Data are presented as means ± SEM.

In order to assess the role of FAK in EGCG-elicited ASM activation, a crucial mechanism in EGCG-induced multiple myeloma cell death [21], U266 multiple myeloma cells were treated with EGCG (10 μM, 3 h) with or without of FAK inhibitor, PF-573228. EGCG upregulated ASM activity and this EGCG-elicited activation of ASM is completely reversed by the pharmacological inhibition of FAK (*p* < 0.05, Student’s *t* test; Figure 3A).

To evaluate the involvement of FAK in EGCG-elicited Akt activation, U266 human multiple myeloma cells were treated with EGCG (10 μM) with or without a FAK inhibitor, PF-573228. Consistent with our previous findings, pharmacological inhibition of FAK significantly neutralized the effect of EGCG on Akt activity (*p* < 0.05, Student’s *t* test; Figure 3B), which is the early mechanism of EGCG-elicited cell death signaling through 67LR activation [25].

Taken together, FAK is involved in the early mechanism that is essential in EGCG-induced Akt/ASM axis in multiple myeloma cells.

As shown Figure 2A,B, EGCG elicited phosphorylation of Src at Tyr 416 in 30 min. In order to assess the role of FAK in EGCG-elicited Src phosphorylation at Tyr 416, U266 cells were treated with EGCG (10 μM, 30 min) in the presence or absence of the FAK inhibitor, PF-573228 (Figure 3C). Western blotting results showed that EGCG treatment induced the phosphorylation of Src at Tyr 416 in 30 min, whereas phosphorylation was not observed in the PF-573228 pretreatment group (Figure 3C).

Taken together, our findings suggested that FAK plays a key role in the EGCG-elicited phosphorylation of Src at Tyr 416, Akt activation, and ASM activation in multiple myeloma cells.

### 2.4. FAK Is the Adaptor Protein of 67LR

**Figure 4:**
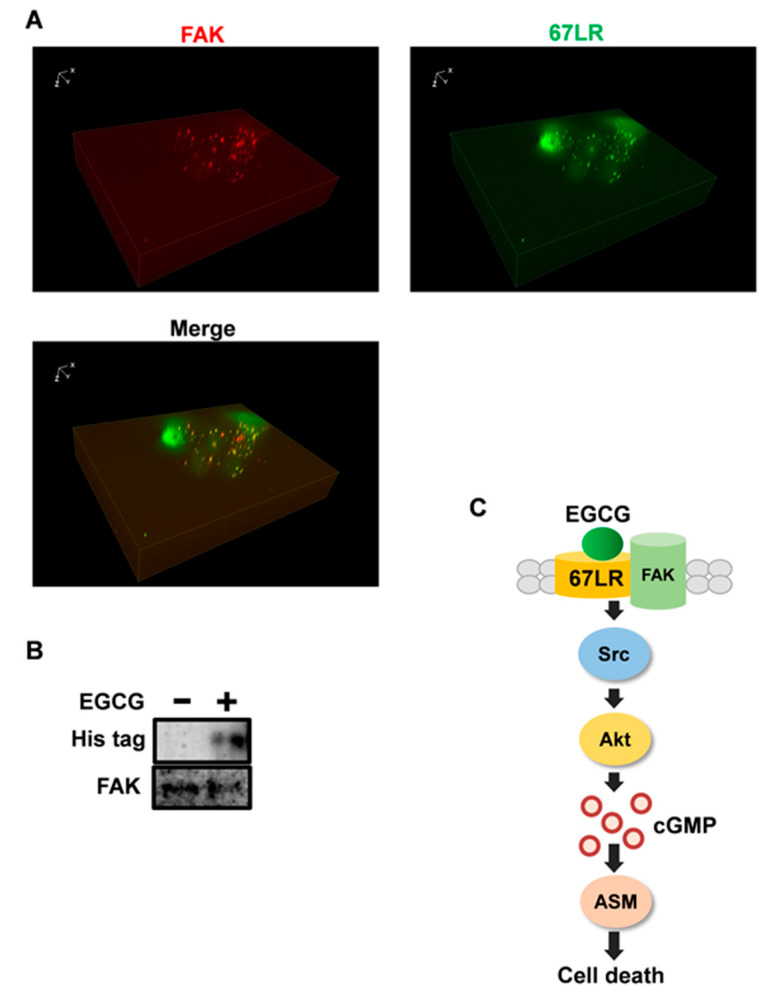
FAK is the adaptor protein of 67LR. (**A**) Immunofluorescence staining for FAK (Red) and 67LR (Green) and Z stack images were determined in HeLa cells using Fluorescence microscopy (×40). (**B**) His-tagged 67LR was immunoprecipitated using the anti-FAK antibody after 30 min EGCG (10 μM) treatment. (**C**) The schematic diagram to summarize the mechanism of EGCG in human multiple myeloma cells. XYZ axis of Z focus image was described above.

FAK is discretely localized to focal adhesions [40] and plays a crucial role in cytoskeletal changes in cells [40]. 67LR is located at the cell membrane; however, to the best of our knowledge, whether it is located at focal adherens junctions has not been elucidated. Since human multiple myeloma cell line U266 cells are not well adherent cells and it is difficult to assess the colocalization of 67LR and FAK in situ. Immunobiological analysis was performed using HeLa cells. Our results indicated that 67LR is mainly located at focal adhesions because FAK is the marker protein showing localization to focal adhesions per se as shown in Figure 4A.

FAK is crucial for Src phosphorylation at Tyr 416 as shown in Figure 3C. 67LR is also involved in the phosphorylation of Src at the same site. Moreover, this protein seems to be colocalized with the focal adhesions; therefore, we hypothesized that there is a direct interaction between 67LR and FAK.

To assess the interaction between 67LR and FAK, cells were transfected with the His-67LR expression vector and treated with EGCG (10 μM, 30 min) and lysates were subjected to immunoprecipitation performed using the anti FAK antibody. Immunoprecipitation results suggested that EGCG induces the interaction between 67LR and FAK as shown in Figure 4B.

Taken together, FAK is involved in the upstream mechanisms of 67LR-dependent signaling.

## 3. Discussion

Several advances have been made in the treatment of multiple myeloma; however, the treatment is still difficult as multiple myeloma is chemoresistant and as patients with multiple myeloma are often old and chemotherapeutic agents have adverse effects on them. Therefore, a novel and modest therapeutic option is urgently required.

Several clinical and preclinical studies have indicated that green tea polyphenol EGCG could be a potential candidate for the treatment of B cell lymphoma and multiple myeloma because it has selective toxicity and is well tolerated [8,20]. However, detail mechanisms are not well understood.

Recent reports indicated that integrin/FAK/Src signal axis plays a crucial role in cancer cell growth [29], motility [41], and invasion [42]. Further, FAK/Src axis is shown to be involved in the oxidative modification of the voltage-gated potassium (K^+^) channel-induced apoptosis and pharmacological inhibition of FAK/Src neutralized the voltage-gated potassium channel oxidation-induced cell death [43]. Consistent with that study, in this study, we found that the FAK/Src axis also plays a key role in the upstream mechanisms, particularly in the activation of ASM, which is a well-known effector in cell death mechanisms. However, the molecular basis of the two paradoxical roles of FAK/Src axis is still unknown and further study is demanded.

We previously reported that 67LR is involved in the anti-inflammatory activity of EGCG through the upregulation of E3 ubiquitin-protein ligase Ring Finger Protein 216 and downregulation of Toll-like receptor 4 (TLR4) accompanied by ubiquitination of TLR4 [34]. In this mechanism, EGCG-induced cGMP upregulation plays a significant role. The present study indicated that Src is involved in the EGCG-elicited cGMP induction; this could act as the early mechanism of EGCG-induced TLR4 downregulation via upregulation of RNF216. We also reported that EGCG/Src/Akt axis was involved in the upregulation of the negative regulator protein, Toll interacting protein (Tollip) in the TLR4 signaling cascade [35]. Our present data indicated that FAK inhibitor attenuated EGCG-induced activation of Akt and phosphorylation of Src. Thus, FAK could be involved in the regulation of EGCG-induced Tollip upregulation in macrophages; however, further studies are needed to elucidate this mechanism.

Recently, we showed that procyanidin C1, the characteristic polyphenol in red wine and cacao, directly bound to 67LR and inhibited growth of melanoma cell accompanied with the cytoskeletal rearrangement [44]. Considering the significance of FAK in cytoskeletal rearrangement, FAK/Src axis could also be involved in the growth inhibitory effect of procyanidin C1 on melanoma cells.

A recent report revealed the importance of FAK in the maintenance of insulin sensitivity in vivo [45]. Clinical data [46,47] and in vivo experiments [48] have indicated that green tea has beneficial effects on insulin sensitivity. These beneficial effects may be exerted via the 67LR/FAK axis.

67LR is a non-integrin laminin receptor with several functions. 67LR has a dual role in cancer including its role in metastasis [18,49]. Further, pathological studies have shown that high 67LR expression is correlated with tumor metastasis and poor prognosis in several types of cancers including colorectal carcinoma [16], and breast cancer [18]. Considering the role of FAK/Src in cell mobility and invasion [29,41,42], those mechanisms could be involved in the negative role of 67LR. Moreover, 67LR also acts as a cancer specific death receptor through activation of the cGMP/ASM axis [25,26,27] and inhibits the growth of melanoma through cytoskeletal rearrangement [19,44]. The two different roles of FAK/Src in cell mobility and apoptosis could explain the paradoxical role of 67LR in cancer cells.

In this study, we demonstrated that proto-oncogene tyrosine-protein kinase Src is an upstream regulator in EGCG-induced Akt activation and that Src is also a crucial mediator in EGCG-induced ASM activation. We showed that EGCG upregulated the phosphorylation of Src at Tyr 416, which plays a crucial role in Src activity. Moreover, we identified FAK as the direct mediator that binds to 67LR in response to EGCG treatment, and 67LR and FAK were found to colocalize. We also showed that FAK is important in the EGCG-induced Src phosphorylation and upregulation of Akt activity.

## 4. Materials and Methods

### 4.1. Cell Culture and Evaluation of Akt Activity

Human multiple myeloma cell line U266 cells were maintained in the Roswell Park Memorial Institute (RPMI) 1640 medium (Fujifilm, Tokyo, Japan) supplemented with 10% fetal bovine serum (Sigma Aldrich, St Louis, MO, USA) and antibiotics (Meiji, Tokyo, Japan) in a humidified incubator with 5% CO_2_ at 37 °C.

Human cervical cancer cell line HeLa cells were maintained in Dulbecco’s modified Eagle medium (Fuji firm) supplemented with 10% fetal bovine serum (Sigma Aldrich) and antibiotics in a humidified condition with 5% CO_2_ at 37 °C. In all cell-based assays, cells were pretreated with indicated inhibitors including 2.5 μM of SKI1 (Abcam, Cambridge, MA, USA) and 1 μM of PF573228 (Sigma Aldrich) for 1 h before EGCG treatment. For evaluation of the effect of EGCG (Sigma Aldrich) on the viability of U266 cells, U266 cells (5 × 10^4^ cells/well) were inoculated into 24-well plates and treated with EGCG for 72 h in RPMI 1640 medium supplemented with 1% fetal bovine serum, 200 units/mL catalase, and 5 units/mL superoxide dismutase (Sigma Aldrich).

For the Akt activity assay, U266 cells were seeded into 24-well plates (1 × 10^6^ cells/well in RPMI 1640 medium) and treated with EGCG for indicated durations and centrifuged. Pellets were treated with lysis buffer and Akt activity was evaluated using the K-LISA^TM^ Akt activity kit (Merck Millipore, Burlington, MA, USA) following the manufacturer’s protocol. Absorbance (450 nm) was assessed using the Envision plate reader purchased from Perkin Elmer and common blank value was subtracted.

For the cGMP assay, U266 cells (2.5 × 10^4^ cells/well; IBMX containing PBS) were seeded into 96 well low volume plates and treated with indicated compounds and incubated at 37 °C and cGMP levels were evaluated using d2 labeled cGMP and K-labeled anti-cGMP antibodies (Perkin Elmer, Waltham, MA, USA). Fluorescence signals were detected using the Envision plate reader purchased from Perkin Elmer using the 665 nm filter 3 h after treatment.

For the antibody masking assay, U266 cells were seeded into 48-well plates (2 × 10^6^ cells/well in RPMI 1640 medium) and treated with EGCG for 30 min with or without the Purified Mouse IgM Isotype Control (eBioscience; San Diego, CA, USA) or Mouse mAb to 67 kDa Laminin Receptor (MLuC5) 20 μg/mL (Abcam, Cambridge, MA, USA).

### 4.2. ASM Activity Evaluation and Western Blotting

For evaluation of the effect of EGCG (Sigma Aldrich) on the activity of ASM in U266 cells, U266 cells (1 × 10^6^ cells/well) were inoculated into 24-well plates and treated with EGCG for 3 h in RPMI 1640 medium and centrifuged. Pellets were treated with lysis buffer (Tris 3.03 g, NaCl 4.38 g, EDTA 0.19 g, NaF 1.05 g, Na_4_P_2_O_7_ 6.70 g, and Triton-X 5 mL, pH 4.5 was adjusted using HCl in a total volume of 500 mL dH_2_O) PMSF and aprotinin were then added. Cell lysates were centrifuged at 12,000× *g*, 10 min at 4 °C, and supernatants were harvested. Substrates for enzyme activity consisted dH_2_O, 1 M sodium acetate (pH 4.5), 10% Triton-X, and 1 mM C12-BODIPY-SM; 34:10:5:1. The enzyme reaction was stopped using chloroform:methanol (2:1 (*v/v*)) stopping buffer. BODIPY^®^-ceramide and BODIPY^®^-SM were evaluated using thin layer chromatography (TLC) using water/chloroform/methanol/(4:65:25, volume ratio) as a solvent. BODIPY^®^-ceramide and BODIPY^®^-SM levels were detected using UV irradiation and analyzed using the FUSION imaging system (Viber-Lourmat, Collégien, France).

For evaluation of the effect of EGCG on phosphorylation of Src in U266 cells, U266 cells (1 × 10^6^ cells/well) were inoculated into plates and treated with EGCG for 30 min in RPMI 1640 medium and then centrifuged. Pellets were treated with lysis buffer (Tris 3.03 g, NaCl 4.38 g, EDTA 0.19 g, NaF 1.05 g, Na_4_P_2_O_7_ 6.70 g, and Triton X 5 mL pH was adjusted to 7.5 using HCl in a total volume of 500 mL dH_2_O) and then PMSF, aprotinin and vanadate activated by H_2_O_2_ were added. Cell lysates were centrifuged at 12000× *g*, 15 min at 4 °C, and supernatants was harvested. Supernatant were denatured using sample buffer (0.057 M Tris-HCl, pH 6.8, 1.8% (*w/v*) sodium dodecyl sulfate, 0.65 M 2-merchaptomethanol, 9.1% glycerol, and 0.02% bromophenol blue).

After performing sodium dodecyl sulfate-polyacrylamide gel electrophoresis (SDS-PAGE), separated protein bands were detected using antibodies including Src (Cell Signaling Technology, Danvers, MA, USA), P-Src (Y527) Rabbit Ab (Cell Signaling Technology), and P-Src (Tyr416) (D49G4) Rabbit mAb (Cell Signaling Technology), each antibody was diluted using 2.5% BSA TTBS (1:3000). Blots were washed with TTBS, blots were incubated with secondary antibodies for 1 h. Bands were detected using FUSION (Viber-Lourmat, Collégien, France). All image processes were performed by Photoshop 5.5 (Adobe, San Jose, CA, USA) and Kyplot 5.0 (KyensLab Inc., Tokyo, Japan).

### 4.3. Immunoprecipitation and Immunofluorescence Staining

For immunofluorescence staining, HeLa cells were seeded on a 35 mm glass bottom dish purchased from Matsunami (Tokyo, Japan) at the density of 3 × 10^4^ cells/mL in 10% FCS DMEM. After 24 h, cells were fixed in 4% paraformaldehyde (Fuji Firm) for 10 min on ice. After washing with PBS, cells were treated with Anti-FAK antibody ((H-1) (Santa Cruz Biotechnology, Dallas, TX, USA) and Rb mAb against 67-kDa Laminin Receptor (EPR8469; abcam) for 1 h on ice. After washing with PBS, cells were incubated with Alexa Fluor^TM^ 555 Fab fragment of goat antimouse IgG (H + L) (Life Technologies Corporation, Eugene, OR, USA) and Alexa Fluor^TM^ 488 Fab fragment of goat antirabbit IgG (H + L) (Life Technologies Corporation). After washing twice with PBS, cells were observed under a BZ-X700 Fluorescent microscope (Keyence, Tokyo, Japan) and Z stack image were obtained following the manufacturer’s protocols.

HeLa cells were transfected with a V5 His 67LR expression vector, seeded at the density of 2 × 10^6^ cells/well in a 12 well plate, and treated with EGCG (10 μM) for 30 min. Cells were then centrifuged and supernatant was removed; the pellet was resuspended using the same lysis buffer as that used for Western blotting. After centrifuging at 12000× *g* for 15 min the supernatant was harvested. Dynabeads Protein G (Thermo Fisher Scientific, Waltham, MA, USA) was treated with an anti-FAK antibody at a concentration of 1 μg/200 μL (diluted with 0.02% Tween 20 containing PBS) for 10 min at room temperature. Cell lysates were added to the pellet of beads and gently mixed for 4 h at 4 °C. After removing the supernatant, beads were washed and transferred to a new tube. Harvest buffer (sample buffer (0.057 M Tris-HCl, pH 6.8, 1.8% (*w/v*) sodium dodecyl sulfate, 0.65 M 2-merchaptomethanol, 9.1% glycerol, and 0.02% bromophenol blue)) and PBS were added at a 1:1 proportion and samples were boiled for 10 min.

### 4.4. Statistical Analysis

All data are presented as means ± S.E.M. Statistical analysis was performed by a Student’s *t* test (one-tail) using the GraphPad Prism software v8 (GraphPad Software, El Camino Real, San Diego, CA, USA). A value of *p* < 0.05 was considered significant.
